# Integrative review of artificial intelligence applications in nursing: education, clinical practice, workload management, and professional perceptions

**DOI:** 10.3389/fpubh.2025.1619378

**Published:** 2025-08-01

**Authors:** Rabie Adel El Arab, Omayma Abdulaziz Al Moosa, Mette Sagbakken, Ahmed Ghannam, Fuad H. Abuadas, Joel Somerville, Abbas Al Mutair

**Affiliations:** ^1^Almoosa College of Health Sciences, Alhasa, Saudi Arabia; ^2^Department of Nursing and Health Promotion, Faculty of Health Sciences, Oslo Metropolitan University, Oslo, Norway; ^3^Department of Computer Science, Princess Sumaya University for Technology, Amman, Jordan; ^4^Department of Community Health Nursing, College of Nursing, Jouf University, Sakaka, Saudi Arabia; ^5^Inverness College, University of the Highlands and Island, Inverness, United Kingdom; ^6^Glasgow Caledonian University, Glasgow, United Kingdom; ^7^Research Center, Almoosa Specialist Hospital, Al-Hasa, Saudi Arabia; ^8^School of Nursing Wollongong, University of Wollongong, Wollongong, NSW, Australia; ^9^Department of Medical-Surgical Nursing, College of Nursing, Princess Nourah Bint Abdulrahman University, Riyadh, Saudi Arabia; ^10^Department of Nursing, Prince Sultan Military College of Health Sciences, Dhahran, Saudi Arabia

**Keywords:** artificial intelligence, nursing education, nursing practice, workload management, clinical decision support, patient monitoring

## Abstract

**Background:**

Artificial Intelligence (AI) is rapidly transforming the nursing profession, presenting significant opportunities and challenges. Despite its promising potential in enhancing nursing education, clinical practice, and operational efficiency, critical barriers related to ethics, workforce adaptation, and humanistic care persist.

**Aim:**

This integrative review systematically evaluates the integration of AI in nursing practice, with a specific focus on nursing education, clinical care, workload management, and professional perceptions.

**Methods:**

Guided by PRISMA 2020 and the SPIDER framework, a thematic synthesis was conducted. Study quality was assessed using the Mixed Methods Appraisal Tool (MMAT), and the risk of bias evaluated through ROBINS-I.

**Results:**

This review encompassed 25 studies, from which six overarching themes emerged.

**Education and training:**

AI-powered simulations and content-creation platforms enriched nursing curricula by presenting realistic clinical scenarios, which consistently yielded deeper student engagement, enhanced case-management performance, and higher satisfaction scores. Learners also reported an increased cognitive load and heightened stress levels when navigating these more complex, AI-driven activities.

**Clinical decision support and monitoring:**

AI-enabled alert algorithms and wearable sensors enabled nurses to detect subtle signs of patient deterioration and fever significantly earlier than conventional methods, supporting timelier clinical interventions. Qualitative feedback from critical-care staff underscores that these automated insights must be balanced with professional judgment to avoid overreliance.

**Rehabilitation and postoperative care:**

In neurosurgical, gynecological, and orthopaedic settings, AI-guided imaging tools and personalized follow-up pathways were linked to smoother recovery trajectories, streamlined follow-up processes and richer patient feedback, and exceptionally high patient satisfaction. Nurses noted that these technologies enhanced the precision of assessments without wholly replacing the need for human touch.

**Workload and workflow management:**

AI systems that automated routine follow-up tasks and generated predictive workload models freed nurses from repetitive, non-clinical duties and offered data-driven insights to inform staffing decisions. These efficiencies allowed nursing teams to devote more time to direct patient care and were associated with reductions in burnout and improved workplace morale.

**Nursing perceptions:**

Across practice settings, nursing students and practicing nurses broadly welcomed AI’s ability to streamline workflows and support decision-making, recognizing its potential to elevate patient care and professional practice.

**Ethical implications:**

Simultaneously, nurses voiced significant ethical concerns—chiefly around safeguarding patient data privacy, mitigating algorithmic bias, and preserving the compassionate, human-centered essence of nursing in an increasingly automated environment.

**Framework and recommendations:**

The Nursing AI Integration Roadmap (NAIIR) was developed, emphasizing transformational education, advanced clinical integration, ethical governance, robust organizational infrastructure, participatory design, and rigorous economic evaluation. This framework offers a structured, ethically informed, and user-centric approach, advocating for AI as complementary to human expertise.

**Conclusion:**

Successfully integrating AI into nursing requires comprehensive strategic planning that addresses educational, clinical, ethical, organizational, participatory, and economic dimensions, reinforcing the core humanistic values of nursing. Of the 25 included studies, 21 were judged at moderate risk of bias; despite this limitation, evidence suggests improvements in critical thinking, learner engagement, and clinical satisfaction across diverse educational and practice settings.

## Introduction

In recent years, the rapid maturation of artificial intelligence (AI) has been recognized as a transformative development with profound implications for healthcare. AI—an umbrella term encompassing machine learning, natural language processing, computer vision, and other advanced computational techniques that enable machines to mimic, support, or enhance human cognitive functions—has matured from an intriguing concept to a suite of tools with demonstrated capacities in pattern recognition, predictive modeling, clinical decision support, and data synthesis (1–3). Across the health sector, AI-driven innovations have begun to harness the massive and heterogeneous data streams generated by electronic health records (EHRs), wearable sensors, imaging modalities, laboratory tests, and patient-reported outcomes (4–6). When thoughtfully designed and implemented, these tools can produce timely, actionable insights that inform diagnosis, risk stratification, treatment planning, resource allocation, and patient engagement (7, 8).

For nursing, AI’s potential extends across multiple domains. Advanced predictive models may help nurses identify early deterioration, reducing morbidity and mortality. Intelligent documentation systems could alleviate administrative burdens, granting nurses more time for direct patient care. AI-guided educational platforms might enrich nursing training, fostering clinical judgment and adaptive reasoning (9–11). In emergency departments, AI has demonstrated superior performance in risk stratification compared to traditional triage scales (12). These AI tools consistently achieve high predictive accuracy (AUC > 0.80) and support the early identification of patients requiring intensive care, providing critical value for nursing teams operating in high-pressure environments (12). Beyond triage, AI tools—particularly large language models (LLMs) like ChatGPT—are reshaping nursing practice through intelligent documentation support, educational automation, and diagnostic assistance. ChatGPT has shown effectiveness in enhancing nursing workflows, facilitating care planning, and reducing cognitive burdens, especially in environments with limited resources (13).

However, the clinical adoption of such technologies is hindered by a persistent translational gap between controlled research settings and the diverse, complex realities of real-world healthcare (14). Methodological limitations, ethical concerns, and workflow misalignment have all slowed AI integration into frontline care (14). Recent findings in neonatal intensive care units highlight how nurses perceive AI as a time-saving and communicative asset—particularly during discharge education—while also voicing concerns over its potential to reduce human interaction and undermine clinical judgment (15).

Despite the promising advancements, the integration of AI into healthcare is accompanied by several challenges. Data privacy and security concerns are paramount, as the utilization of vast amounts of patient data necessitates stringent measures to protect sensitive information (16). Ethical considerations also arise, particularly regarding the extent to which AI should influence clinical decision-making and the potential for biases inherent in AI algorithms (17). Moreover, the swift advancement of technological innovation requires nursing professionals to consistently adapt and enhance their skills to effectively utilize AI tools (18). This necessitates comprehensive training programs and ongoing professional development to ensure that nurses remain proficient in using AI technologies and can critically evaluate their applications.

The integration of AI in nursing education necessitates a reevaluation of existing curricula to incorporate AI literacy and competencies. Given AI’s multifaceted impact on nursing education and practice, conducting an integrative review is essential to synthesize existing evidence, identify best practices, and highlight areas requiring further research (19). Existing reviews have mapped high-level themes—AI literacy needs, ethical and social safeguards, and equitable deployment—yet remain conceptual, synthesizing secondary literature without direct evaluation of hands-on educational tools, participatory co-design methods, or economic feasibility (20). Likewise, integrative analyses of primary studies have demonstrated concrete clinical and operational gains—improvements in diagnostic accuracy, early detection, and workload reduction—but have not systematically addressed AI-driven pedagogy, governance frameworks, or cost–benefit considerations (21). This review defines three AI modalities— (1) machine learning–based predictive models, (2) large language models (e.g., GPT-4), and (3) wearable sensor technologies—and examines their unique applications in nursing education and clinical practice. The present review uniquely bridges these domains—uniting AI-enhanced education, decision support, workflow optimization, ethical governance, participatory design, and economic evaluation—into one cohesive, evidence-based roadmap for sustainable, human-centered AI integration in nursing.

## Aim

Aim: To conduct an integrative synthesis of existing evidence on AI integration in nursing, articulating a unifying framework that bridges education, practice, and organizational domains.

### Objectives

1. Analyze AI’s impact on nursing education, including skill development and simulation.
2. Assess AI-driven interventions in clinical care and patient monitoring.
3. Evaluate AI’s effects on workload management and professional perceptions.

## Study design

This integrative review was meticulously conducted in accordance with the Preferred Reporting Items for Systematic Reviews and Meta-Analyses (PRISMA) 2020 guidelines, ensuring transparency, reproducibility, and comprehensive reporting (22). The review employed a thematic analysis approach to synthesize qualitative and quantitative data from included studies, enabling the identification of key themes concerning the impact of AI in nursing (23). We conducted an inductive thematic synthesis, iteratively coding until saturation.

To capture the full spectrum of qualitative, quantitative, and mixed-method evidence on AI in nursing, we adopted the SPIDER (Sample, Phenomenon of Interest, Design, Evaluation, Research type) framework rather than the quantitatively focused PICO model (24). PICO’s emphasis on “Comparison” and narrowly defined outcomes tends to overlook rich experiential and implementation data, whereas SPIDER was specifically developed to guide searches in mixed-methods and qualitative syntheses. The detailed SPIDER criteria are outlined in Table 1.

**Table 1:** SPIDER framework for eligibility criteria.

Component	Details
Sample	Nursing professionals, including registered nurses, nurse educators, and nursing students.
Phenomenon of Interest	Integration of artificial intelligence (AI) technologies in nursing practice and education.
Design	Quantitative, qualitative, and mixed-methods studies.
Evaluation	Impact on nursing education, clinical decision support systems, patient monitoring tools, rehabilitation and postoperative care, workload and workflow management, and nurses’ perceptions and attitudes toward AI.
Research Type	Empirical studies, including randomized controlled trials, cohort studies, cross-sectional studies, and qualitative studies.

### Inclusion and exclusion criteria

To ensure the relevance and quality of the included studies, specific inclusion and exclusion criteria were established based on the SPIDER framework (see Table 2 for inclusion and exclusion criteria).

**Table 2:** Inclusion and exclusion criteria.

Inclusion	Exclusion
Nursing professionals (registered nurses, nurse educators, nursing students).	Other healthcare professionals (e.g., physicians, pharmacists).
Studies examining the integration of AI technologies in nursing practice or education.	Studies focusing on AI in other healthcare domains without specific relevance to nursing.
Impact on nursing education, clinical decision support, patient monitoring, workload management, and perceptions/attitudes toward AI.	Studies not reporting on the specified outcomes.
Quantitative, qualitative, and mixed-methods empirical studies.	Reviews, meta-analyses, editorials, commentaries, grey literature and non-empirical studies.
Published in English.	Published in languages other than English.

### Information sources and search strategy

A comprehensive literature search was conducted across various electronic databases to ensure a complete collection of relevant studies. The databases searched included PubMed, CINAHL, Scopus, Web of Science, and IEEE Xplore. Articles were searched across selected databases from their inception up to September 2024, followed by an additional search in March 2025 to capture new publications released after September 2024.

Additionally, the reference lists of included studies and pertinent reviews were hand-searched to identify any additional relevant studies. Grey literature was excluded to maintain the focus on peer-reviewed empirical research. Consistent with our SPIDER framework, only studies involving nursing students or practicing nurses were eligible; studies focusing solely on patient outcomes were excluded.

Complete, field-tagged search strategies for PubMed, CINAHL, Web of Science, Scopus, and IEEE Xplore are provided in Supplementary Appendix 1.

### Selection process

The records identified through comprehensive database searches were imported into Rayyan, a specialized systematic review screening platform (25). To ensure methodological rigor and minimize bias, two authors (RA, FA) conducted the screening process. Any discrepancies were resolved through consensus discussions, or, if needed, by consulting a third reviewer (JS). The protocol for resolving discrepancies involved detailed discussions to reach consensus, ensuring that inclusion decisions were unbiased and based on predefined criteria. Initially, titles and abstracts were evaluated against established inclusion and exclusion criteria. Following this, the full texts of potentially eligible studies were obtained and carefully examined for final inclusion based on the set criteria.

**Figure 1:**
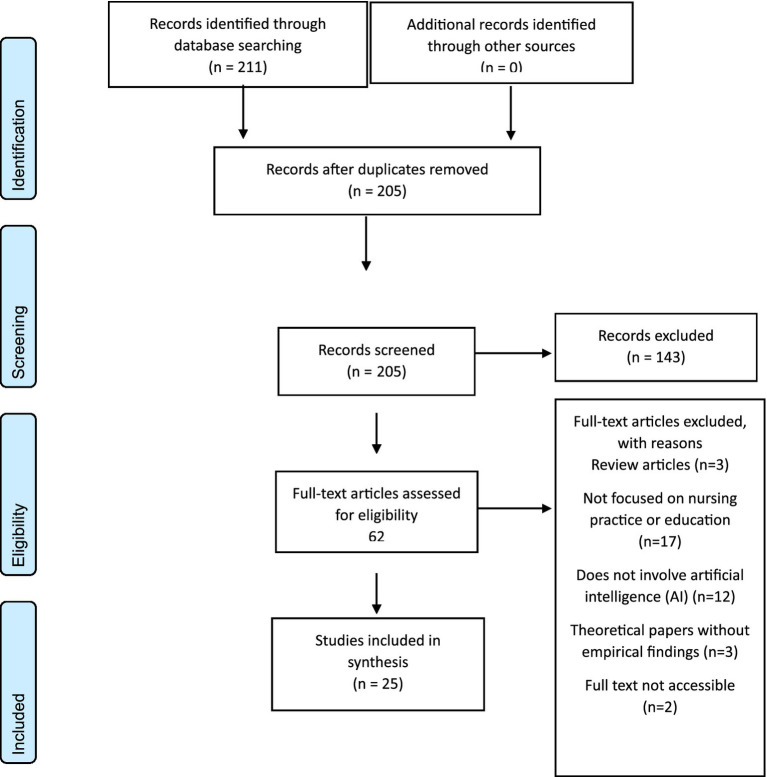
PRISMA flow diagram.

The study selection process is detailed in the PRISMA flow diagram (Figure 1).

### Data extraction

Data extraction was conducted independently by authors RA and JS utilizing a standardized data extraction form to ensure consistency and accuracy. The standardized form included predefined variables and was piloted to ensure reliability. The extracted variables encompassed study characteristics (e.g., author(s), year of publication, country, and study design), population details (e.g., sample size, demographic information, and nursing roles), AI technologies employed, and measured outcomes (e.g., clinical outcomes, operational efficiency metrics, and staff well-being indicators). Additionally, key findings were systematically documented.

To ensure data integrity and reliability, any discrepancies in the extracted data were resolved through deliberation or by consulting a third reviewer. Each study is identified by its first author followed by “et al.” Only first authors are listed in this summary; co-first and co-last authors were each counted individually in our analyses, and all middle (non–first/non–last) authors were excluded. For multicenter or multi-department studies, each contributing department was counted once per paper (not once per author). No gender-assignment analysis was performed for the authors of the included studies, as this was beyond the scope of our integrative objectives. Consequently, we have no gender breakdown or unclassified cases to report, and no associated misclassification bias to assess.

## Results

### Study selection and characteristics

This integrative review encompassed 25 studies published between 2020 and 2024 across regions including North America, Asia, Europe, Africa, the Middle East, and South America. The research designs comprised randomized controlled trials, observational studies, as well as qualitative and mixed method approaches. Populations varied from patients with acute and chronic conditions to nursing professionals and students. Interventions involved AI-driven tools such as clinical decision support systems, educational simulations, workload management applications, and mobile apps for stress reduction. Measured outcomes included clinical metrics (e.g., ICU transfers, readmission rates), educational effectiveness, nurse well-being (e.g., burnout), and attitudes toward AI. Detailed characteristics of each study are provided in Appendix 2.

### Risk of bias

The authors assessed the risk of bias in all 25 included studies using the ROBINS-I tool (Risk Of Bias in Non-randomized Studies of Interventions) (26). Four studies were deemed to have a low risk of bias (27–30), while the remaining 21 were classified as having a moderate risk of bias (31–51). The main contributors to moderate bias were insufficient adjustment for confounders like baseline health status, demographic variables, and previous AI exposure, dependence on subjective or self-reported outcome measures, and selective reporting stemming from the lack of pre-specified protocols or registrations.

Selection bias was generally low, attributed to well-defined inclusion and exclusion criteria in most studies; however, a few studies employed convenience sampling, which may limit generalizability. Interventions were typically well-defined, adherence to intervention protocols was high, and missing data were appropriately managed using robust statistical methods. These findings indicate that the majority of studies should be interpreted with caution, highlighting the need for more rigorous methodological approaches and comprehensive control of confounding factors in future research (see Appendix 3).

### Overall quality assessment of included studies

The methodological quality of the 25 studies included in this integrative review was meticulously evaluated using the Mixed Methods Appraisal Tool (MMAT) (52). The assessment revealed that most of these studies demonstrated high methodological quality, with 22 out of 25 meeting all five MMAT criteria. These high-scoring studies consistently presented clear and relevant research questions, employed appropriate and diverse sampling strategies, utilized representative samples, and applied validated and reliable measurement tools. Additionally, most studies achieved acceptable response and follow-up rates, enhancing the credibility and reliability of their findings.

However, two studies received a score of four out of five due to minor limitations. Specifically, Alruwaili et al. (40) and Sommer et al. (32) were noted to have partial representativeness of their samples and potential nonresponse biases, respectively. Despite these minor limitations, the studies offered valuable insights and employed robust methodologies (32, 40). Common strengths across the included studies included the clear articulation of research objectives and the rigorous use of validated measurement instruments.

Overall, the high methodological quality of most of the included studies enhances the reliability of this integrative review’s conclusions regarding the application of artificial intelligence in nursing and healthcare. The robust study designs, valid measurements, and comprehensive data collection methods employed across these studies provide a solid foundation for drawing meaningful conclusions and identifying best practices in AI integration. Nevertheless, the identified limitations highlight areas for improvement, such as increasing sample diversity and ensuring detailed reporting of randomization procedures, to further strengthen the evidence base in this rapidly evolving field (see Appendix 4).

### Thematic synthesis

The analysis revealed six themes that encapsulate the integration of AI technologies in nursing practice: 1. AI in Nursing Education and Training, 2. AI in Clinical Decision Support and Patient Monitoring, 3. AI in Rehabilitation and Postoperative Care, 4. AI in Nursing Workload and Workflow Management, and 5. Nurses’ Perceptions and Attitudes Toward AI. Additionally, 6. ethical and safety implications emerged as a critical overarching theme influencing all areas of AI integration. Table 3 presents a summary of the findings aligned with the research objectives, detailing key themes and their corresponding findings.

**Table 3:** Summary of findings aligned with objectives.

Objective	Key themes and subthemes	Key findings
Assess the Impact of AI Applications Across Nursing Practice	AI in Nursing Education and Training	AI-assisted simulations improved engagement, satisfaction, and critical thinking. Enhanced educational materials through AI tools increased quality and understandability.
	AI in Clinical Decision Support	AI systems improved diagnostic accuracy, early detection, and decision-making. Wearable AI devices enhanced patient monitoring, reducing ICU transfers and hospitalizations. Concerns over overreliance on AI affecting clinical expertise.
	Patient Monitoring Tools	AI-supported remote monitoring tools facilitated timely interventions and improved patient outcomes. Devices like wearable thermometers detected issues earlier than traditional methods.
	AI in Rehabilitation and Postoperative Care	AI-enhanced diagnostics and personalized interventions improved recovery outcomes. Streamlined follow-ups with AI reduced time and resource demands but lacked comprehensive patient feedback compared to human-led care.
	AI in Nursing Workload and Workflow Management	AI automated administrative tasks, optimized staffing, and predicted workloads. AI-based classifiers and predictive tools enhanced efficiency and allowed nurses to focus on patient care.
Examine Nursing Professionals’ Perceptions, Attitudes, and Experiences	Nurses’ Perceptions and Attitudes Toward AI	Nurses viewed AI as a tool to enhance efficiency and support decision-making. AI was recognized for reducing administrative burdens and improving care quality. Younger nurses were more receptive to adopting AI technologies.
	Concerns and Barriers	Concerns included job displacement. Apprehensions over depersonalized care and erosion of critical thinking skills. Resistance to change and knowledge gaps hindered adoption.
Identify Ethical and Safety Implications of AI in Nursing Practice	Ethical and Safety Implications of AI in Nursing Practice	Concerns over extensive data collection and potential misuse were prevalent. Robust frameworks for data protection and algorithm auditing were recommended.
	Algorithmic Bias and Fairness	Regular auditing of AI systems are seen required to identify and mitigate biases. To prevent disparities in care, it is essential to ensure equitable outcomes.
	Preservation of Human Touch	Nurses emphasized maintaining empathy and the human element in care. AI should complement nursing judgment and patient interaction, rather than replace them.
	Impact on Professional Roles	AI has the potential to shift nurses’ roles towards overseeing and validating AI-generated outputs. Concerns were raised over the potential erosion of professional identity and critical thinking skills due to overreliance on AI.

### AI in nursing education and training

AI technologies have become instrumental in transforming nursing education by enhancing skill acquisition, case management, and patient education. Studies indicate that AI-assisted interactive simulations provide an engaging and satisfying learning environment. For instance, research by Simsek-Cetinkaya & Cakir (34) demonstrated that while traditional patient simulations were more effective in improving breast self-examination skills, AI-assisted simulations significantly boosted student satisfaction and engagement. Similarly, Akutay et al. (27) found that AI-supported case analysis improved students’ ability to prioritize diagnoses and apply clinical concepts, although it did not significantly affect satisfaction levels. Moreover, Saatçi et al. (28) highlighted that AI-assisted preparation of patient education materials enhanced their understandability and quality, suggesting that AI can effectively augment educational strategies by fostering critical thinking and communication skills among nursing students. These findings collectively suggest that AI can play a pivotal role in modernizing nursing education, despite some challenges related to student anxiety and performance metrics.

### AI in clinical decision support and patient monitoring

AI technologies have markedly influenced clinical decision-making and patient monitoring, leading to improved diagnostic accuracy and patient outcomes. In emergency nursing settings, AI applications have been shown to enhance the quality of life and emotional stability of patients with chronic conditions, as evidenced by Hong et al. (33). Wearable AI devices, such as the iThermonitor WT705 evaluated by Liu et al. (35), have demonstrated superior accuracy and early detection capabilities compared to traditional methods, thereby facilitating timely interventions. Burns et al. (30) implemented an early-illness detection algorithm on a medical ward, with nursing staff receiving and acting on algorithm-generated alerts; this collaborative workflow was associated with a marked reduction in ICU transfers. However, Hassan and El-Ashry’s qualitative study (50) highlights that—and despite real-time AI data’s potential to strengthen decision-making—an overreliance on automated alerts risks diminishing nurses’ critical thinking and clinical judgment if not carefully balanced with human oversight. Furthermore, Rony et al. (38) emphasize the importance of preserving the human touch in clinical assessments, advocating for AI to serve as a supportive tool rather than a replacement for human judgment. Overall, AI integration in clinical decision support and patient monitoring offers substantial benefits in terms of efficiency and patient care, if it is implemented in a manner that complements rather than supplants human expertise.

### AI in rehabilitation and postoperative care

The application of AI in rehabilitation and postoperative care has shown significant promise in enhancing patient outcomes and optimizing nursing workflows. Studies by Zhang et al. (30) and Bian et al. (31) demonstrate that AI-enhanced diagnostic tools, such as AI-enhanced MRI and AI-assisted follow-up systems, improve diagnostic precision and reduce resource consumption. These technologies facilitate more personalized and effective nursing interventions, leading to better patient recovery and reduced complication rates. For instance, AI-enhanced MRI in pelvic floor muscle rehabilitation for rectal cancer patients resulted in more precise diagnoses and improved anal function (31), while AI-assisted follow-up systems in orthopedic postoperative care reduced time and resource expenditure while maintaining high follow-up rates and enhancing feedback quality (51). These findings underscore the potential of AI to streamline rehabilitation and postoperative care processes, thereby enhancing both efficiency and patient quality of life. However, it is noted that while AI can enhance the efficiency of these processes, the depth of feedback may sometimes be less comprehensive compared to human interaction, highlighting the need for a balanced integration of AI and human oversight.

### AI in nursing workload and workflow management

AI-driven tools have significantly impacted nursing workload and workflow management by automating routine tasks and providing predictive insights for resource allocation. Rosa et al. (44) developed an AI-driven classifier model that accurately predicts nursing workload based on electronic patient records, thereby reducing manual effort and allowing nurses to concentrate more on patient care and complex decision-making. Similarly, Chen et al. (47) evaluated an “Internet + Hospital-to-Home (H2H)” nutritional nursing model integrated with AI-enhanced CT imaging for chronic kidney disease patients, demonstrating improvements in nutritional outcomes, biochemical indicators, and quality of life. Brom et al. (49) developed a CART-based model that predicted 30-day hospital readmissions (11.2% rate); when its outputs are integrated into nurse-led discharge planning, they enable targeted follow-up and education for high-risk patients. Zhao et al. (29) further demonstrate AI’s role in chronic disease management by pairing an AI-enhanced ultrasound imaging algorithm with comprehensive nursing interventions, yielding significant improvements in renal artery resistance and reduced complication rates. Together, these studies show how embedding predictive and imaging-based AI tools within nursing workflows can streamline care transitions, reduce workload, and bolster patient management—thereby enhancing operational efficiency and safety.

### Nurses’ perceptions and attitudes toward AI

The successful integration of AI technologies in nursing practice is heavily influenced by the perceptions and attitudes of nursing professionals. Surveys and qualitative studies reveal a generally positive outlook towards AI’s potential to enhance efficiency and support clinical decisions. However, there are notable concerns regarding knowledge gaps, trust, and the ethical implications of AI adoption. Sommer et al. (32) found that while a significant proportion of nurses’ view AI favorably for its efficiency-enhancing capabilities, only a quarter felt adequately knowledgeable about AI technologies. Additionally, female nurses exhibited more negative attitudes towards AI compared to their male counterparts, citing concerns about depersonalization of care, job displacement, and the risk of AI errors due to biased training data. An explorative, sequential mixed-methods study by Seibert et al. (48) identified significant barriers to AI integration, including data challenges, ethical issues, and handling task complexity. The study also emphasized facilitators like participatory design and strong data infrastructure. A qualitative study by Hassan and El-Ashry (50) and Rony et al. (38) further emphasizes the need for maintaining human oversight and ensuring that AI serves as a supportive tool rather than a replacement for human judgment. These findings indicate that while there is optimism about AI’s potential to enhance nursing practice, addressing knowledge gaps, building trust, and mitigating ethical concerns are essential for fostering positive attitudes and facilitating effective AI integration within the nursing workforce.

### Ethical and safety implications

Integrating AI into nursing practice introduces various ethical and safety considerations that require careful management to ensure responsible and equitable implementation. Data privacy and security emerge as paramount concerns, with studies by Hong et al. (33) and Rony et al. (38) highlighting significant apprehensions regarding the extensive collection and use of personal health data by AI systems. Nurses advocate for clear guidelines and robust frameworks to govern the ethical implementation of AI, ensuring patient data confidentiality and compliance with data protection regulations.

Algorithmic bias and fairness are critical ethical concern that may result in unequal treatment outcomes. Racine et al. (42) emphasized the necessity of regularly auditing AI algorithms to identify and mitigate biases, thereby ensuring fairness and preventing discrimination in patient care. Additionally, excessive reliance on AI systems poses another ethical challenge, potentially eroding nurses’ critical thinking skills and clinical expertise. Dependence on AI recommendations could diminish nurses’ ability to identify subtle or nuanced patient needs that require human intuition and empathy.

The impact of AI on job roles and professional identity is also a significant concern. As Alruwaili et al. discuss, maintaining patient autonomy and trust in AI-assisted care is vital (40). Further, ensuring transparency in AI systems and preserving the human element in patient interactions are essential for fostering trust and supporting patient autonomy in care decisions.

## Discussion

This integrative review, synthesizing findings from 25 studies, alongside insights from related research, demonstrates that the thoughtful integration of artificial intelligence (AI) into nursing practice can enhance multiple facets of care. Evidence extends to nursing education, clinical decision support, patient monitoring, rehabilitation and postoperative care, workload management, and the evolving perceptions and attitudes of nurses towards AI. Overall, the findings underscore AI’s potential to enhance efficiency, patient outcomes, and workforce sustainability. The findings also stress the need for rigorous ethical oversight, transparent governance, comprehensive training, and participatory design to preserve the humanistic essence of nursing.

### AI in nursing education and training

In the realm of nursing education, AI-enhanced tools have shown potential for creating more dynamic learning environments and enhancing critical thinking skills. Simsek-Cetinkaya & Cakir (34) that although AI-assisted simulations did not outperform standard simulations in improving breast self-examination skills, they substantially boosted student engagement and satisfaction. Similarly, Akutay et al. (27) showed improved prioritization of nursing diagnoses through AI-supported case analyses, and Saatçi et al. (28) found that AI-assisted preparation of patient education materials improved their understandability, actionability, and overall quality.

These advances align with findings from the Clinician Champions Program (53), which effectively increased participants’ AI knowledge and confidence via interactive learning, reflective exercises, and a capstone project. By emphasizing equity, diversity, and flexible access—key principles embodied in frameworks like IDEA (inclusion, diversity, equity, and accessibility)—such programs ensure that the emerging nursing workforce is not only technically competent but also ethically and culturally sensitive.

Overcoming knowledge gaps, alleviating learner anxiety, and ensuring equitable resource distribution continue to be major challenges, but the trajectory is clear: AI has the potential to enhance nursing education, equipping practitioners for increasingly data-driven clinical environments.

### AI in clinical decision support and patient monitoring

Clinical decision support and patient monitoring represent areas where AI’s impact is particularly evident. Multiple studies in this review indicate that AI-driven systems can improve early detection of patient deterioration, potentially refining decision-making, and foster proactive interventions.

These findings resonate with those reported by Gallo et al. (54), who found that an AI deterioration model reduced care escalation events, and Mau et al. (55), who observed that AI-augmented early warning systems improved responsiveness and lowered the incidence of severe events.

Similarly, Choudhury et al. (56) highlighted that AI-enabled decision support enhances error detection, patient stratification, and drug management. Moreover, predictive modeling insights from Xue et al. (57) confirmed that machine learning algorithms can accurately forecast postoperative complications, potentially guiding real-time interventions and risk mitigation. However, as Alanazi (57) cautions, the integration of AI in clinical workflows raises important questions regarding data quality, privacy, transparency, and ethical standards—concerns that must be diligently addressed.

### AI in rehabilitation and postoperative care

In rehabilitation and postoperative care, AI offers avenues for more personalized and efficient care pathways. Zhang et al. (31) found that AI-enhanced imaging improved diagnostic precision in pelvic floor rehabilitation for rectal cancer patients, facilitating more targeted nursing interventions and better functional outcomes. Bian et al. (51) demonstrated that AI-assisted postoperative follow-up systems in orthopedic care could reduce resource demands while maintaining comprehensive patient feedback.

These advancements align with the predictive potential demonstrated by Xue et al. (58), indicating that AI-driven risk stratification can inform both rehabilitation planning and postoperative surveillance. Yet, the importance of maintaining empathetic, trust-based relationships must not be understated. While AI-driven efficiencies can streamline patient follow-up, they must be balanced against the need for human warmth, emotional support, and reassurance.

### AI in nursing workload and workflow management

AI’s capacity to predict workload, optimize staffing, and reduce administrative burdens may also improve workforce sustainability and patient safety. Rosa et al. (44) developed an AI-based classifier for predicting nursing workload, potentially mitigating burnout and improving resource allocation. In chronic disease management, Chen et al. (47) integrated AI-enhanced imaging and nutritional assessment into a home-based care model, improving clinical indicators, patient satisfaction, and operational efficiency. Complementing these findings, Hazarika et al. (59) highlighted that AI can enhance productivity, foster interdisciplinary collaboration, and improve provider satisfaction.

However, these advancements depend on robust IT infrastructures, transparent algorithmic methodologies, and inclusive design processes. As Alanazi (57) and Hazarika (59) emphasize, without adequate ethical and regulatory frameworks, challenges related to data bias, accountability, and algorithmic fairness remain unresolved.

### Nurses’ perceptions, attitudes, and ethical considerations

Nurses’ perceptions and attitudes towards AI critically influence its adoption. Sommer et al. (32) observed optimism about efficiency gains and apprehensions about depersonalization. Rony et al. (38) and Hassan et al. (50) echoed these concerns, emphasizing that AI should not undermine nurses’ professional autonomy, empathy, or critical thinking. Instead, AI should support evidence-based practice, relieve nurses from routine tasks, and allow more time for nuanced patient care.

Addressing these concerns demands ethical safeguards, careful data stewardship, and transparent decision-making processes. Choudhury et al. (56) highlighted the need for standardized benchmarks and ongoing model validation. The Clinician Champions Program (53) and participatory design approaches (48) serve as models for building trust and acceptance: by including nurses and other stakeholders in the development and refinement of AI tools, healthcare systems can ensure that technology aligns with clinicians’ values, respects patient dignity, and promotes equitable care delivery. Moreover, the convergence of clinical AI systems and patient-controlled platforms (e.g., GPOC) highlights new governance challenges—and opportunities—for nurses acting as data stewards and care partners. A robust, internationally harmonized regulatory latticework is therefore essential to safeguard both patient autonomy and data privacy as these hybrid model’s scale (60).

The promise of AI—enhanced patient outcomes, increased operational efficiencies, and enriched educational experiences—is evident, yet fully realizing its potential depends on sustained research, ethical oversight, and continuous professional development.

### Strengths and limitations

Our review’s key strengths lie in its comprehensive scope—drawing on five major databases (PubMed, CINAHL, Scopus, Web of Science, and IEEE Xplore) and synthesizing evidence from qualitative, quantitative, and mixed-methods studies to provide a richly nuanced portrait of AI integration in nursing. It addresses highly topical issues—ethical governance, educational innovation, and operational efficiency—ensuring the findings are immediately relevant to both researchers and practitioners. Given the moderate risk of bias observed in most studies, our conclusions are presented with caution; we recommend future high-quality randomized trials with rigorous methodology to confirm these preliminary insights. Finally, the application of two complementary appraisal tools (MMAT for overall methodological quality and ROBINS-I for bias in non-randomized studies) demonstrates rigorous, structured evaluation of study validity, lending confidence to our conclusions.

This integrative review has several limitations. We further acknowledge that our decision to exclude grey literature—specifically doctoral theses, conference abstracts, and preprints—may introduce a publication bias favoring fully published, peer-reviewed studies. This approach can over-represent mature, positive, or methodologically robust findings while under-capturing early-stage work, null or negative results, and emerging insights often first presented at conferences or in dissertation form. Consequently, our review may paint a somewhat optimistic picture of AI’s integration in nursing, potentially underestimating implementation challenges, preliminary failures, or novel applications reported outside traditional journals. Future syntheses should consider incorporating grey literature to ensure a more balanced appraisal of both established and nascent evidence. Additionally, the variability in study designs and methodologies among the included studies may limit the comparability of results. The majority of studies assessed had a moderate risk of bias, which necessitates cautious interpretation of the overall findings.

We acknowledge that 21 of the 25 included studies were rated as having a moderate risk of bias under ROBINS-I, principally due to incomplete adjustment for confounders and non-randomized designs. Formal, quantitative sensitivity analyses (e.g., leave-one-out or meta-regression) were not feasible given the heterogeneity of study designs, outcomes, and reporting formats. Instead, we conducted a qualitative “low-risk cross-check”—examining whether the four low-risk studies alone would uphold our principal themes and the six pillars of the NAIIR framework. These low-risk studies did indeed reflect the same directional trends in educational engagement, diagnostic precision, workload optimization, and ethical safeguards, albeit with more conservative effect estimates. Consequently, while our conceptual roadmap and thematic conclusions are reinforced across both moderate- and low-risk studies, the precise magnitude of reported benefits should be interpreted with appropriate caution. We recommend that future research employ standardized outcome measures and robust confounder adjustment to enable formal sensitivity and subgroup analyses, thereby strengthening the quantitative precision of AI’s impact in nursing. We did not stratify studies by clinical domain (for example, hepatology vs. other gastroenterology topics) and therefore cannot assess whether AI–nursing research activity reflects the true clinical case volumes or economic burden.

### Recommendations

The successful integration of artificial intelligence (AI) into nursing requires a strategic, multifaceted approach addressing educational, clinical, ethical, infrastructural, participatory, and economic dimensions. Nursing education should incorporate foundational AI competencies systematically, employing AI-enhanced simulations and interactive learning platforms to cultivate critical thinking and technical proficiency. Structured professional development programs and micro-credentialing must be established to maintain workforce alignment with evolving AI technologies, thus sustaining high-quality patient care and operational excellence.

Clinical integration demands the seamless embedding of AI-driven decision-support and patient-monitoring tools into existing workflows. These technologies, including predictive analytics, wearable monitoring devices, and automated administrative systems, should be deeply integrated within electronic health records (EHRs) to enhance real-time decision-making, patient safety, and workflow efficiency. Predictive workload management systems must be prioritized to optimize staffing, reduce burnout, and enhance nurses’ capacity for direct patient engagement and complex clinical reasoning.

Robust ethical oversight and governance frameworks must underpin AI implementations in nursing. Comprehensive protocols addressing data privacy, informed consent, transparency, and fairness in algorithmic decision-making should be rigorously enforced. Regular audits and interdisciplinary governance committees involving nurses, ethicists, and technologists are essential to uphold ethical standards and maintain patient trust.

To support AI integration, healthcare institutions should substantially invest in robust technological infrastructure, secure interoperable systems, and specialized technical support teams. Strategic change management processes, involving clear leadership, interdisciplinary collaboration, and cultural adaptation strategies, are critical to address resistance and promoting organizational resilience and agility in adapting to technological advancements.

Centralizing participatory design practices will ensure that AI solutions are user-centric, clinically relevant, and practically beneficial. Continuous engagement of frontline nursing staff, educators, and patients through structured feedback loops and iterative refinements will enhance the usability, acceptance, and effectiveness of AI tools, aligning them closely with real-world clinical environments.

Rigorous economic evaluation and sustainability assessments must become a core component of AI integration strategies. Comprehensive cost–benefit analyses, return-on-investment studies, and economic forecasting will provide essential insights to guide resource allocation, ensuring economic feasibility and sustained organizational commitment to AI initiatives.

Our integrative review captured studies published between early 2020 and late 2024, revealing a distinct acceleration in AI–nursing research coinciding with the COVID-19 pandemic. To maintain currency with this rapidly evolving field, we recommend transitioning to a living review model, with scheduled search updates (e.g., biannually or quarterly) across both traditional databases and preprint servers (such as medRxiv and SSRN). Incorporating automated keyword alerts and expanding search terms to capture novel AI approaches will ensure that future reviews continually assimilate the burgeoning evidence base from 2025 onward.

### Implications

The integration of artificial intelligence into nursing practice and education reshapes care delivery, workforce management, and institutional operations. Clinically, AI enables more accurate diagnostics, proactive interventions, personalized care plans, continuous patient monitoring, shorter hospital stays, and enhanced safety. In education, it fosters interactive, adaptive learning environments that strengthen critical thinking and clinical skills. Administratively, AI-driven automation and predictive staffing ease workloads, boost job satisfaction, and mitigate burnout. Robust ethical frameworks and governance protocols safeguard transparency, fairness, patient autonomy, and human oversight while addressing risks of depersonalization. Strategic implementation requires significant investment in infrastructure, interoperability, data security, and sustained technical support. Financial analyses ensure that AI adoption is economically viable and aligned with organizational policies.

Future research must rigorously evaluate AI’s long-term effects on clinical outcomes, patient satisfaction, workforce resilience, and economic returns through longitudinal and cost-effectiveness studies. Development and validation of adaptive, learner-centered educational platforms should be prioritized, alongside in-depth examination of ethical safeguards for data privacy, bias mitigation, and algorithmic transparency. Investigations into effective interdisciplinary collaboration models will help tailor AI tools to real-world nursing needs. Domain-specific studies—particularly in geriatric care, mental health, and critical care—are needed to assess targeted applications. Finally, incorporating patient perspectives on AI-enhanced nursing will be essential to foster trust and optimize care experiences.

### Nursing AI integration roadmap (NAIIR): a comprehensive strategic framework

The advent of AI represents a watershed moment for the nursing profession, promising unprecedented enhancements in clinical outcomes, operational efficiency, education quality, ethical rigor, and healthcare sustainability. Recognizing this transformative potential, the Nursing AI Integration Roadmap (NAIIR) is proposed as a robust, evidence-based strategic framework designed to guide stakeholders comprehensively through the complex journey of AI integration. The NAIIR strategically addresses key dimensions critical to effective and sustainable AI implementation: educational transformation, clinical integration, ethical governance, organizational preparedness, participatory innovation, and rigorous economic assessment.

#### Transformational education and competency development

NAIIR prioritizes educational reform to foster a nursing workforce proficient in AI literacy, critical analysis, and ethical decision-making. Nursing curricula should systematically incorporate foundational AI competencies, including machine learning principles, predictive analytics, ethical implications, and data privacy considerations. Interactive, AI-enhanced simulation training and micro-credentialing pathways will provide hands-on experience, fostering critical thinking and clinical judgment. Structured continuous professional development programs will ensure ongoing alignment of nurse competencies with rapid technological advancements, preparing a resilient, informed, and adaptable workforce.

#### Advanced clinical integration and workflow enhancement

This pillar advocates for a seamless, practical integration of AI tools within clinical workflows to elevate patient care delivery, clinical efficiency, and nurse autonomy. AI-driven decision-support platforms, integrated within electronic health records (EHRs), will facilitate precise risk stratification, early detection, and improved diagnostic accuracy. Sophisticated AI-enabled patient monitoring solutions, including wearable devices and remote surveillance technologies, will provide continuous, real-time patient assessments. Automation of administrative responsibilities and intelligent predictive workforce management systems will enable nurses to refocus their valuable time on direct patient care, complex clinical decisions, and compassionate interactions, thereby significantly mitigating burnout risks and enhancing professional satisfaction.

#### Ethical excellence, transparency, and governance

Recognizing AI’s ethical implications, NAIIR emphasizes a rigorous governance framework ensuring responsible, transparent, and equitable AI deployment. Robust protocols addressing data privacy, security, and informed consent are mandatory. Continuous algorithm audits and fairness assessments must be institutionalized to proactively identify and mitigate biases. Ethical training embedded within nursing education and professional development will reinforce the importance of maintaining human oversight, preserving nurses’ critical judgment, and safeguarding patient-centered care practices. Active, interdisciplinary governance involving ethicists, clinicians, and technologists will maintain ethical standards and patient trust.

#### Robust organizational infrastructure and strategic interoperability

AI integration necessitates resilient, sophisticated organizational infrastructure supporting seamless interoperability across healthcare platforms. Investment in advanced technological infrastructure, secure and scalable data networks, and interoperable IT systems will underpin AI’s effective and sustainable use. Dedicated technical teams providing ongoing training, support, and troubleshooting will facilitate smooth operational transitions and minimize clinical disruptions. Comprehensive change management, supported by interdisciplinary leadership, clear communication strategies, and cultural adaptation initiatives, will foster organizational agility, resilience, and innovation.

#### Participatory design and continuous quality improvement

A fundamental principle of NAIIR is the active, ongoing engagement of frontline nurses, educators, and patients in the co-creation and iterative refinement of AI technologies. Adopting participatory design methodologies ensures AI solutions are user-centric, practical, and aligned with clinical realities. Continuous, structured feedback mechanisms—such as digital dashboards, satisfaction surveys, and regular stakeholder workshops—will provide actionable insights to guide iterative enhancements. This dynamic, inclusive approach guarantees sustained relevance, usability, and effectiveness of AI implementations.

#### Rigorous economic evaluation and sustainable investment

Financial sustainability underpins successful, long-term AI adoption. Comprehensive economic evaluations, including rigorous cost–benefit analyses, return-on-investment (ROI) assessments, and scenario-based forecasting, must guide decision-making processes. Strategic prioritization of AI projects will ensure that resource allocation is justifiable, economically viable, and strategically aligned with healthcare goals. Regular financial assessments will ensure ongoing economic sustainability, fostering confidence among healthcare administrators, stakeholders, and policymakers.

### Structured implementation roadmap

The implementation roadmap begins with a comprehensive assessment and strategic planning phase, where baseline competencies, technological capacities, and economic considerations are thoroughly evaluated alongside inclusive stakeholder engagement to identify priorities and potential barriers. Following this foundational phase, targeted pilot initiatives will be deployed within selected clinical and educational settings to validate AI tools and gather detailed quantitative and qualitative feedback, including economic feasibility assessments. Subsequently, the framework supports a system-wide deployment phase, featuring institution-wide roll-out of validated AI technologies, reinforced by continuous training, established ethical protocols, and robust infrastructural readiness. Continuous performance monitoring and iteration constitute the next phase, characterized by real-time tracking of clinical outcomes, user satisfaction, and economic metrics to inform ongoing refinements. Finally, the roadmap culminates in policy formulation and regulatory alignment, emphasizing collaboration with policymakers and advocacy for comprehensive ethical guidelines and robust policy frameworks to support the long-term sustainable integration of AI within nursing practice.

## Conclusion

This integrative review comprehensively evaluates AI integration into nursing practice, highlighting significant enhancements in education, clinical care, ethical oversight, workforce management, organizational efficiency, and economic sustainability. The Nursing AI Integration Roadmap (NAIIR) offers a strategic framework, addressing key domains essential to successful AI integration. This structured approach ensures effective educational transformations, seamless clinical implementations, rigorous ethical governance, robust infrastructural support, participatory innovations, and comprehensive economic evaluations. The recommendations, implications, and research directions outlined herein provide a high-impact, actionable guide for stakeholders to achieve meaningful, sustainable integration of AI within nursing, ultimately advancing global healthcare delivery standards.

## Data Availability

The original contributions presented in the study are included in the article/Supplementary material, further inquiries can be directed to the corresponding author.
