# Supplementary material for: Integrative review of artificial intelligence applications in nursing: education, clinical practice, workload management, and professional perceptions
Source: Front Public Health. 2025 Aug 1;13:1619378. doi: 10.3389/fpubh.2025.1619378 (PMC12354398; doi:10.3389/fpubh.2025.1619378)
Supplement: Supplementary file 1 [file Table_1.DOCX]

**Appendix 1. Full database-specific search strategies**

| **Database** | **AI Terms** | **Nursing Terms** | **Clinical/Edu Terms** | **Date Limits** | **Language** |
| --- | --- | --- | --- | --- | --- |
| **PubMed** | “Artificial Intelligence”[MeSH] OR “AI”[tiab] OR “machine learning”[MeSH] OR “deep learning”[tiab] OR “natural language processing”[tiab] OR “computer vision”[tiab] OR “predictive modeling”[tiab] OR “data mining”[tiab] | “Nursing”[MeSH] OR nurse*[tiab] OR “nurse educator”[tiab] OR “nursing student”[tiab] | “Clinical Decision Support Systems”[MeSH] OR “decision support”[tiab] OR “patient monitoring”[tiab] OR wearable sensor*[tiab] OR simulation[tiab] OR “workload management”[tiab] OR burnout[tiab] OR “job satisfaction”[tiab] OR perception*[tiab] OR attitude*[tiab] | (“1900/01/01” – “2024/09/30”) OR (“2025/03/01” – “2025/03/31”) | English |
| **CINAHL** (EBSCO) | MH “Artificial Intelligence+” OR AI OR “machine learning” OR “deep learning” OR “natural language processing” OR “computer vision” OR “predictive modeling” OR “data mining” | MH “Nursing+” OR nurs* OR “nurse educator” OR “nursing student” | MH “Clinical Decision Support Systems” OR “decision support” OR “patient monitoring” OR wearable sensor* OR simulation OR “workload management” OR burnout OR “job satisfaction” OR perception* OR attitude* | Publication Year 1900–2024; 2025 (Mar 2025 update) | English |
| **Web of Science** | TS=(“Artificial Intelligence” OR AI OR “machine learning” OR “deep learning” OR “natural language processing” OR “computer vision” OR “predictive modeling” OR “data mining”) | TS=(nurs* OR “nurse educator” OR “nursing student”) | TS=(“decision support” OR “patient monitoring” OR wearable sensor* OR simulation OR “workload management” OR burnout OR “job satisfaction” OR perception* OR attitude*) | PY=1900–2024 OR 2025 | English |
| **Scopus** | TITLE-ABS-KEY(“Artificial Intelligence” OR AI OR “machine learning” OR “deep learning” OR “natural language processing” OR “computer vision” OR “predictive modeling” OR “data mining”) | TITLE-ABS-KEY(nurs* OR “nurse educator” OR “nursing student”) | TITLE-ABS-KEY(“decision support” OR “patient monitoring” OR wearable sensor* OR simulation OR “workload management” OR burnout OR “job satisfaction” OR perception* OR attitude*) | PUBYEAR ≤ 2024 OR = 2025 | English |
| **IEEE Xplore** | (“Abstract”:“Artificial Intelligence” OR “Index Terms”:“Artificial Intelligence” OR Abstract:AI OR Abstract:“machine learning” OR Abstract:“deep learning” OR Abstract:“natural language processing” OR Abstract:“computer vision” OR Abstract:“predictive modeling” OR Abstract:“data mining”) | (“Abstract”:nurs* OR Abstract:“nurse educator” OR Abstract:“nursing student”) | (“Abstract”:“decision support” OR “Abstract”:“patient monitoring” OR “Abstract”:“wearable sensor*” OR Abstract:simulation OR “Abstract”:“workload management” OR Abstract:burnout OR “Abstract”:“job satisfaction” OR Abstract:perception* OR Abstract:attitude*) | Publication_Year:1900–2024 OR 2025 | English |
