# Supplementary material for: Integrative review of artificial intelligence applications in nursing: education, clinical practice, workload management, and professional perceptions
Source: Front Public Health. 2025 Aug 1;13:1619378. doi: 10.3389/fpubh.2025.1619378 (PMC12354398; doi:10.3389/fpubh.2025.1619378)
Supplement: Supplementary file 2 [file Table_2.DOCX]

Appendix 2: Characteristics of studies included in the systematic review

| Study ID | Year | Country | Study Design | Population/Setting | Intervention | Outcomes Measured | Key Findings | Limitations |
| --- | --- | --- | --- | --- | --- | --- | --- | --- |
| Burns et al. | 2022 | Canada | Prospective interventional quality improvement study | Conducted on a 40-bed medical ward in a 300-bed tertiary care hospital. Included 273 patients (110 control, 163 treatment) randomly admitted to the medical ward. | The study implemented a novel software program designed to detect acute illness early using an algorithm that analyzed vital signs. | Transfers from the medical ward to the intensive care unit (ICU).  Ward nurses were the primary users | ICU transfers were significantly reduced in the treatment group (3 vs. 14 in the control group), reflecting an 85.54% relative risk reduction. | The study was limited to one medical ward with a small sample size, potentially affecting generalizability |
| Zhao et al. | 2022 | China | Prospective interventional study | Conducted on 44 patients diagnosed with diabetic kidney disease (DKD) | Comprehensive nursing intervention (CNI) was implemented for Group B. The impact of CNI was evaluated using ultrasound imaging enhanced by an intelligent algorithm | Renal artery resistance index (RI). | The RI of patients in Group B decreased significantly after intervention (0.63 ± 0.06) compared to Group A (0.68 ± 0.07, *p* < 0.05). | The study involved a small sample size, potentially limiting generalizability. |
| Brom et al. | 2020 | United States | Retrospective observational study | Conducted at a 300-bed urban academic medical center, focusing on 2,165 admissions to medical services | Utilized a Classification and Regression Tree (CART) machine learning algorithm applied to electronic health record (EHR) data to identify patients at high risk of 30-day readmission | All-cause 30-day hospital readmission rates.  Nursing handoffs & discharge workflows incorporated the CART risk scores to guide nursing prioritization and patient education. | The 30-day readmission rate was 11.2% (n=242). | The study relied on retrospective data, limiting causal inferences |
| Alruwaili et al. | 2024 | Saudi Arabia | Descriptive cross sectional | 220 registered nurses from three governmental hospitals in the Jouf region, with at least one year of clinical experience. | Assessment of nurses' awareness and attitudes toward AI tools in clinical practice using an online survey with validated instruments. | Awareness of AI, attitudes toward AI, and demographic factors influencing these attitudes. | Nurses displayed moderate AI awareness; 58.2% had used AI in healthcare. Positive attitudes were more common among younger and undergraduate-educated nurses, while females and older nurses showed conservative attitudes. Concerns included AI's impact on privacy, job roles, and error potential. | Limited generalizability due to setting, cross-sectional design, and convenience sampling. Future studies should explore qualitative insights and longitudinal impacts |
| Hong et al. | 2021 | China | Randomized Controlled Trial (RCT) with Sub-Analyses | 447 COPD patients in emergency care; sub-cohort of 101 and 29 patients. Highlight nurse‐delivered telemedicine. | AI-based medical intervention (web-based knowledge exercises, telemedicine) | Quality of life at 4 and 12 months, hospitalization rate, length of stay | Significant improvement in quality of life after 12 months, reduced hospitalization rates, shorter hospital stays in AI groups. No improvement at 4 months. | High dropout rate due to internet issues; limited generalizability; small sample size in sub-cohorts. |
| Rony et al. | 2024 | Bangladesh | Descriptive Qualitative Study | 23 nursing professionals at three tertiary hospitals in Dhaka | Perspectives on AI integration in nursing care | Themes: AI readiness, patient outcomes, training, ethical considerations, human-tech balance | Nurses expressed optimism toward AI for enhancing care but highlighted training, ethical, and workflow concerns. | Limited to a small sample size from a single region; no patient or AI developer perspectives included. |
| Leon et al. | 2023 | Brazil | Observational Study | 28 nursing students undergoing maternal-child clinical simulation | Analysis of emotions using Artificial Intelligence (AI) to assess facial expressions, tone of voice, and speech content | Emotions observed during simulation: valence (positive/negative), control, obstruction, and task performance | AI revealed oscillation between positive and negative emotions; students experienced stress but recognized simulation as valuable for learning. | Limited to a single educational institution; lack of validated cases, absence of personality screening, and no comparison with standard teaching. |
| Bian et al. | 2020 | China | Exploratory Quantitative and Qualitative Study | 270 orthopedic patients (AI-assisted follow-up) and 2656 patients (manual follow-up). Emphasize nursing follow‐up role. | AI-assisted postoperative follow-up system compared to manual follow-up | Telephone connection rate, follow-up rate, feedback collection rate, time spent, feedback composition | AI-assisted follow-up achieved similar effectiveness to manual follow-up with higher feedback rates and lower time costs. Feedback depth in AI group was limited. | Short probation period for AI system, no integration with additional follow-up methods like chatbots |
| Xu et al. | 2022 | China | Quasi-Experimental Study | 86 intracranial aneurysm patients (43 experimental, 43 control) undergoing craniotomy clipping | AI-enhanced cerebral angiography (Otsu method) combined with perioperative nursing intervention | Surgical outcomes, length of stay, complications, quality of life, and nursing satisfaction | AI-enhanced ICGA significantly improved treatment outcomes, reduced complications, and enhanced quality of life compared to standard care. | Small sample size, short postoperative follow-up, and limited to a single cent |
| Cho et al. | 2024 | South Korea | Single-Arm Trial | 300 nurses experiencing burnout | AI-based tailored mobile intervention using "Nurse Healing Space" app | Burnout levels, job stress, stress response, usability, user satisfaction | Significant reduction in burnout, job stress, and stress responses; satisfaction increased with optimized AI recommendations. | Lack of control group, limited generalizability due to specific sample, ongoing algorithm optimization. |
| Jiang et al. | 2022 | China | Comparative Experimental Study | 116 patients with ovarian endometriosis | MRI diagnosis using AI-based FCM algorithm and comprehensive nursing intervention | MRI diagnostic accuracy, Dice coefficient, sensitivity, specificity, nursing satisfaction, adverse reactions | AI-FCM algorithm significantly improved diagnostic accuracy, Dice, and specificity; comprehensive nursing reduced adverse reactions and improved satisfaction. | Limited to a single hospital; long-term follow-up on patient outcomes not included. |
| Marcuzzi et al. | 2023 | Norway | Randomized Clinical Trial | 294 patients with neck and/or low back pain referred to specialist care | Self-management app deployed within nurse-led rehabilitation clinics, with nurses onboarding patients, monitoring adherence, and integrating app data into individualized care plans. | Musculoskeletal health (MSK-HQ), pain intensity, pain-related disability, and quality of life at 3 and 6 months | AI-based app did not significantly improve musculoskeletal health over usual care or web-based intervention; modest impact on perceived global effect. | Low engagement with the app; no significant long-term improvement; generalizability limited to similar care settings. |
| Liu et al. | 2020 | China | Prospective Comparative Study | 526 surgical patients in surgical wards at a university hospital. Highlight reduced manual checks | Continuous temperature monitoring using AI-based wearable device (iThermonitor WT705) compared to mercury thermometer | Temperature accuracy, precision, fever detection, comfort, and feasibility of continuous monitoring | iThermonitor showed acceptable accuracy (mean bias: 0.03°C), earlier fever detection (4.35 hours), and higher peak temperature recording. | Accuracy reduced in hypothermic or underweight patients; results limited to axillary temperature only. |
| Du et al. | 2022 | China | Prospective Experimental Study | 64 diabetic nephropathy patients receiving home nursing intervention | PDCA (Plan-Do-Check-Action) home nursing strategy evaluated with fMRI under AI-based FCM clustering algorithm | Nursing effectiveness, satisfaction scores, service quality, and quality of life over 12 months | PDCA nursing significantly improved curative effects, patient satisfaction, and quality of life compared to routine care. AI-based FCM improved fMRI analysis accuracy and efficiency. | Small sample size; no consideration of long-term impacts on renal and glycemic indicators. |
| Yin et al. | 2022 | China | Prospective Controlled Study | 60 postpartum women with pelvic organ prolapse (POP-Q grade I–II) | Pelvic floor rehabilitation training (PFMT, biofeedback, electrical stimulation) evaluated via AI-processed ultrasound imaging | Levator ani muscle thickness, perineal hiatus diameter, pelvic floor muscle strength, and image quality | Rehabilitation training significantly improved pelvic floor muscle strength and reduced prolapse severity. AI-enhanced ultrasound imaging provided clearer results and enhanced image analysis. | Small sample size; short follow-up period; limited to mild and moderate pelvic organ prolapse cases. |
| Simsek et al. | 2023 | Turkey | Comparative Intervention Trial | 103 nursing students learning breast self-examination (BSE) | AI-assisted interactive screen-based simulation (AI-AISBS) vs. standard patient simulation (SPS) | BSE skill performance, satisfaction with simulation, and state anxiety levels | SPS was more effective for improving BSE skills. AI-AISBS increased student satisfaction but also anxiety levels. | Small sample size; short-term evaluation; limited to a single nursing school. |
| Seibert et al. | 2023 | Germany | Exploratory Sequential Mixed Methods Study | Stakeholders in AI and nursing care (21 workshop participants, 14 interviewees, 53 survey respondents, 80 datathon participants) | Exploration of needs, challenges, and opportunities for AI in nursing care | Needs for AI applications, prioritization of scenarios, and success criteria for research projects | Identified needs for AI solutions in care assessment, planning, decision support, education, and resource management. Barriers include regulatory issues, data quality, and ethical concerns. | Limited to German context; short study duration; lack of data saturation due to exploratory nature. |
| Racine et al. | 2024 | Canada and UK | Multisite Qualitative Study | 20 health care professionals (HCPs) and 20 parents of preterm infants in NICUs at 2 tertiary care hospitals | Use of AI for pain monitoring in neonatal intensive care units (NICU) | Perceptions of AI for pain monitoring, ethical considerations, implementation requirements | HCPs and parents recognized AI's potential for improving neonatal pain care but emphasized its role as a supportive tool rather than a replacement for clinical judgment. Concerns included ethical issues, emotional distress, and integration challenges. | Limited to two NICUs in high-resource countries; perspectives were hypothetical due to lack of prior exposure to AI tools. |
| Chen et al. | 2022 | China | Randomized Controlled Trial | 120 patients with CKD stages 3-5 using hospital-to-home (H2H) care | AI-based “Internet + H2H” nutritional nursing model combined with CT imaging | Anthropometry, laboratory indicators, renal perfusion (BF), patient satisfaction, and NRS 2002 score | AI-enhanced H2H model significantly improved nutritional status, biochemical markers, renal blood flow, and patient satisfaction compared to standard care. | Single-center study; no separate analysis for CKD stages; results may not generalize beyond the study setting. |
| Eman Hassan et al. | 2024 | Egypt | Interpretive Phenomenological Analysis (IPA) | 10 critical care nurse leaders in ICUs across four hospitals | Exploration of AI integration in ICU care, including its impact on practice, roles, trust, and ethical considerations | Perceptions of AI’s role in decision-making, workload, trust-building, collaboration, and ethical dilemmas | AI supports task automation and data analysis but raises concerns about overreliance, workflow challenges, ethical bias, and patient autonomy. Successful integration requires transparency, training, and trust-building. | Small sample size, limited to leadership roles; bedside nurses’ perspectives were not captured. |
| Sommer et al. | 2024 | Germany | Cross-sectional Online Survey | 114 nurses from various care settings in Bavaria | Survey on nurses' perceptions, knowledge, and experiences of AI in nursing care | AI knowledge, areas of application, attitudes (positive/negative), and barriers | 25.2% of nurses self-identified as AI experts; 65.7% viewed AI as an opportunity for reducing workload and enhancing care. Concerns included job loss, costs, and lack of AI knowledge. | Small sample size, limited to Bavaria, potential sampling bias, and lack of longitudinal data. |
| da Rosa et al. | 2024 | Brazil | Retrospective Observational Study | 43,871 patient assessments using Perroca's Patient Classification System (PCS). Emphasize nurse‐manager use of workload model | Development of an AI-based predictive nursing workload classifier using machine learning (Random Forest algorithm) | Accuracy, sensitivity, F1-score, ROC-AUC curve for workload prediction and key workload variables | AI-based predictive model achieved 72% accuracy (AUC = 82%). Key workload predictors included bed baths, fall risk, and wound care protocols. Model automation can optimize care planning and staff management. | Single-center study; lower performance for semi-intensive and intensive care classes; results may not generalize to other settings. |
| Akutay et al. | 2024 | Turkey | Randomized Controlled Trial | 188 third-year nursing students in a university setting | AI-supported case analysis lecture vs. traditional instructor-led case lecture for THA (Total Hip Arthroplasty) | Case management performance, satisfaction, focus, interest, and nursing diagnoses | AI-supported case analysis improved case management performance and knowledge test scores significantly. Satisfaction, focus, and interest levels were similar between AI and control groups. | Single-center study, limited to one nursing case (THA), no long-term retention assessment, and inability to interact with AI avatars. |
| Saatçi et al. | 2024 | Turkey | Randomized Controlled Trial | 180 first-year nursing students preparing patient education materials | AI tools (ChatGPT, Gemini, Canva) for preparing patient education materials vs. traditional methods (books, journals, websites) | Understandability, actionability, and quality of patient education materials | AI-supported materials significantly improved understandability (75.36 vs. 57.57), actionability (34.71 vs. 17.56), and quality (GQS: 3.65 vs. 2.23) compared to traditional methods. | Single topic ("Rational Drug Use"), limited to one university, no patient evaluation of materials, and absence of qualitative exploration of students' experiences. |
| Zhang et al. | 2022 | China | Randomized Controlled Study | 88 gastrointestinal rectal cancer patients undergoing anus-preserving surgery | Comprehensive pelvic floor muscle rehabilitation exercises compared to Kegel exercises | Anorectal function (score), quality of life (EORTC QLQ-CR29), MRI imaging quality with AI enhancement | Comprehensive pelvic floor training significantly improved anorectal function and quality of life scores. AI-enhanced MRI provided clearer imaging for diagnosis and assessment. | Single-center study, small sample size, short intervention duration (3 months), no long-term follow-up. |
