# Supplementary material for: Integrative review of artificial intelligence applications in nursing: education, clinical practice, workload management, and professional perceptions
Source: Front Public Health. 2025 Aug 1;13:1619378. doi: 10.3389/fpubh.2025.1619378 (PMC12354398; doi:10.3389/fpubh.2025.1619378)
Supplement: Supplementary file 4 [file Table_4.DOCX]

Appendix 4: Risk of Bias

| Authors (year) | Bias due to Confounding | Bias in Selection of Participants | Bias in Classification of Interventions | Bias due to Deviations from Intended Interventions | Bias due to Missing Data | Bias in Measurement of Outcomes | Bias in Selection of Reported Results | Overall Risk of Bias |
| --- | --- | --- | --- | --- | --- | --- | --- | --- |
| Burns et al. (2022) | Low | Low | Low | Low | Low | Low | Low | Low |
| Zhao et al. (2022) | Low | Low | Low | Low | Low | Low | Low | Low |
| Brom et al. (2020) | Moderate | Low | Low | Low | Low | Low | Low | Moderate |
| Zhang & Wang (2022) | Moderate | Low | Low | Low | Low | Low | Moderate | Moderate |
| Saatçi et al. (2024) | Low | Low | Low | Low | Low | Low | Moderate | Low |
| Akutay et al. (2024) | Low | Low | Low | Low | Low | Low | Moderate | Low |
| Rosa et al. (2024) | Moderate | Low | Low | Low | Low | Moderate | Moderate | Moderate |
| Sommer et al. (2024) | Moderate | Moderate | Low | Low | Low | Moderate | Moderate | Moderate |
| Hassan & El-Ashry (2024) | Moderate | Moderate | Low | Low | Low | Moderate | Moderate | Moderate |
| **Chen et al. (2022)** | Moderate | Low | Low | Low | Low | Moderate | Moderate | **Moderate** |
| Racine et al. (2024) | Moderate | Moderate | Low | Not applicable | Low | Moderate | Moderate | Moderate |
| Seibert et al. (2023) | Moderate | Moderate | Not Applicable | Not Applicable | Low | Moderate | Moderate | Moderate |
| Simsek-Cetinkaya & Cakir (2023) | Moderate | Low | Low | Low | Low | Moderate | Moderate | Moderate |
| Yin & Wang (2022) | Moderate | Low | Low | Low | Low | Moderate | Moderate | Moderate |
| Du et al. (2022) | Moderate | Low | Not Applicable | Not Applicable | Low | Moderate | Moderate | Moderate |
| Liu et al. (2020) | Moderate | Low | Low | Not Applicable | Low | Moderate | Moderate | Moderate |
| Marcuzzi et al. (2023) | Moderate | Low | Low | Low | Low | Moderate | Moderate | Moderate |
| Jiang et al. (2022) | Moderate | Low | Low | Not Applicable | Low | Moderate | Moderate | Moderate |
| Cho et al. (2024) | Moderate | Moderate | Not Applicable | Not Applicable | Low | Moderate | Moderate | Moderate |
| Xu et al. (2022) | Moderate | Low | Low | Low | Low | Moderate | Moderate | Moderate |
| Bian et al. (2020) | Moderate | Low | Low | Low | Low | Moderate | Moderate | Moderate |
| Ponce de Leon et al. (2023) | Moderate | Moderate | Not Applicable | Not Applicable | Low | Moderate | Moderate | Moderate |
| Rony et al. (2024) | Moderate | Moderate | Not Applicable | Not Applicable | Low | Moderate | Moderate | Moderate |
| Hong et al. (2021) | Moderate | Moderate | Low | Low | Low | Moderate | Moderate | Moderate |
| Alruwaili et al. (2024) | Moderate | Moderate | Not Applicable | Not Applicable | Low | Moderate | Moderate | Moderate |
